# Cross-Modal Collaboration and Robust Feature Classifier for Open-Vocabulary 3D Object Detection

**DOI:** 10.3390/s25020553

**Published:** 2025-01-19

**Authors:** Hengsong Liu, Tongle Duan

**Affiliations:** The 54th Research Institute, China Electronics Technology Group Corporation, College of Signal and Information Processing, Shijiazhuang 050081, China; liuhengsong@sjtu.edu.cn

**Keywords:** 3D object detection, multi-sensor fusion, zero-shot learning, autonomous driving

## Abstract

The multi-sensor fusion, such as LiDAR and camera-based 3D object detection, is a key technology in autonomous driving and robotics. However, traditional 3D detection models are limited to recognizing predefined categories and struggle with unknown or novel objects. Given the complexity of real-world environments, research into open-vocabulary 3D object detection is essential. Therefore, this paper aims to address two key issues in this area: how to localize and classify novel objects. We propose Cross-modal Collaboration and Robust Feature Classifier to improve localization accuracy and classification robustness for novel objects. The Cross-modal Collaboration involves the collaborative localization between LiDAR and camera. In this approach, 2D images provide preliminary regions of interest for novel objects in the 3D point cloud, while the 3D point cloud offers more precise positional information to the 2D images. Through iterative updates between two modalities, the preliminary region and positional information are refined, achieving the accurate localization of novel objects. The Robust Feature Classifier aims to accurately classify novel objects. To prevent them from being misidentified as background or other incorrect categories, this method maps the semantic vectors of new categories into multiple sets of visual features distinguished from the background. And it clusters these visual features based on each individual semantic vector to maintain inter-class separability. Our method achieves state-of-the-art performance on various scenarios and datasets.

## 1. Introduction

**Figure 1:**
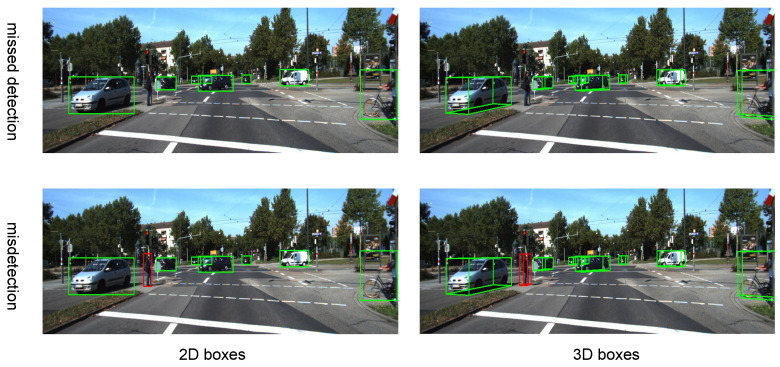
The misdetection and missed detection issues faced by traditional object detection. Green means that it is detected correctly while red means that it is misdetected as a cyclist. It can be seen that pedestrians, as a novel object, cannot be correctly recognized, which inevitably impacts the overall detection performance.

Three-dimensional object detection is a crucial and challenging task in applications like autonomous driving and robotics, requiring precise and robust environmental perception across various scenarios [1]. As Figure 1 shows, traditional object detection models are limited to recognizing predefined categories, struggling to identify novel or unseen objects in complex environments [2]. It can be observed that when the training set only annotates the car and cyclist classes while neglecting the pedestrian class, our detection performance is suboptimal. In practical applications, we may not always have the ability to annotate all the objects to be detected in the training set. To address this limitation, we explore open-vocabulary 3D object detection [3], where the model, trained on a limited set of categories, can detect and recognize new categories not present in the training data. This approach enhances the model’s understanding of 3D space, improving its overall scene perception [4].

In the problem of open-vocabulary 3D object detection, the key challenge is how to localize and classify novel objects using only annotations from a limited set of base categories [5,6]. To date, there are very few works in the literature addressing this challenging problem. Some approaches borrow ideas from 2D image-text pre-training [7] and leverage text embeddings to classify novel 3D objects. Although some progress [8] has been made in recognizing novel objects, these methods face two main issues. First, the models rely solely on point cloud data, and the use of a single sensor source limits the accuracy and robustness in localizing novel objects [9]. Second, the classification of novel objects remains problematic. While these models have moved beyond predefined categories, they often misclassify novel objects as background or incorrectly categorize them. Consequently, the detectors in these works struggle to generalize effectively to novel objects [10].

In this paper, we propose Cross-modal Collaboration and Robust Feature Classifier to address the challenges of novel object localization and classification, respectively. Our proposed algorithm specifically targets the limitations of existing methods. Cross-modal Collaboration fully leverages data from multiple sensors, achieving more accurate localization compared to using only LiDAR data. The Robust Feature Classifier is designed to tackle the difficulty of classifying novel objects, particularly the issues of misclassification as background or incorrect categorization as other classes. The detailed mechanisms of these approaches are described below.

The Cross-modal Collaboration framework is designed to leverage multimodal and multi-sensor data during training, containing 2D photos and 3D point cloud information. Compared to models that rely solely on LiDAR sensors, a critical improvement in our model is that multi-sensor information works together to make the detection more accurate. This approach coordinates localization between the 2D and 3D modalities, sharing semantic and feature knowledge across them. When the 2D modality performs localization, the 3D modality assists by projecting the 2D detection box onto the 3D point cloud, quickly narrowing down the approximate target region. This allows the rich semantic knowledge from the 2D detector to be transferred into the 3D domain to assist in discovering new objects. Similarly, when the 3D modality is localizing, the 2D modality collaborates by utilizing the rich point cloud data to refine the bounding box with greater precision. This geometric knowledge helps mitigate the limitations of the 2D detection images, which lack direct 3D supervision.Through continuous collaborative localization between the 2D and 3D modalities, each modality refines the detection results of the other, leading to a positive feedback loop that enhances detection outcomes.

The Robust Feature Classifier introduces a novel zero-shot object classification framework designed to address the feature synthesis challenges in real-world application cases. In the detection process, novel objects may be mistakenly classified as background due to insufficient features or misclassified as other categories due to feature similarity. To avoid these errors, our classifier is designed to learn robust region features. We propose a visual feature diverging component that can expand the semantic vector of a single class into a set of visual features, distinguishing the object from the background. To further prevent misclassification into other categories, we introduce an inter-class difference preserving component, which utilizes real visual samples from various object categories to constrain the separability of the synthesized visual features. These two components of the Robust Feature Classifier work together to enhance the accuracy of object recognition.

In order to verify the effectiveness of our method, we conduct experiments on the widely used SUN RGB-D [11], nuScenes [8] and KITTI [12] datasets. They encompass a variety of indoor and outdoor scenes. We perform ablation experiments and use different baseline networks to compare ours, such as Det-PointCLIP, OV-3DET, and CoDA. The results demonstrate that our method achieves competitive results in a variety of scenarios, and outperforms the state of the art. The key contributions of this paper can be summarized as follows:

- We propose a unified open-vocabulary 3D detector contains Cross-modal Collaboration and Robust Feature Classifier. It takes advantage of the benefits of multi-sensors and excels in detecting objects of any class across diverse scenes, thus greatly enhancing the perception of real scenes in 3D vision.
- The Cross-modal Collaboration use the synergy of camera and LiDAR to pinpoint novel objects. This collaboration mechanism is superior compared with other multimodal fusions. The Robust Feature Classifier learns robust features for more accurate object classification.
- Comprehensive experiments on three datasets, including SUN RGB-D, nuScenes and KITTI, demonstrate the effectiveness of the proposed approach. Notably, our method achieves comparable performance to state-of-the-art approaches whether it is an indoor or outdoor scene.

## 2. Related Work

### 2.1. 3D Object Detection

Three-dimensional object detection focuses on predicting object categories and oriented three-dimensional bounding boxes (BBoxes) for scenes. This field has seen rapid advancements, largely driven by industries such as autonomous driving, where understanding and interpreting the environment in three dimensions is critical. Early foundational methods, such as PointNet [13], directly processed raw point cloud data, laying the groundwork for subsequent innovations in 3D object detection. Building on this foundation, VoteNet [14] introduced a novel voting mechanism that significantly enhanced detection accuracy, improving the precision of object localization and classification. MLCVNet [15], another key contribution, integrated multi-level feature learning and contextual data to improve the robustness of the detection process, addressing challenges such as noise and occlusions in point cloud data.

In recent years, transformer-based architectures and multimodal techniques have gained increasing attention in the field [16]. Transformer models leverage self-attention mechanisms to effectively capture long-range dependencies in point cloud data [17], allowing for improved global feature consistency and more accurate object recognition. In addition, multimodal techniques, which fuse data from multiple sources, such as images and point clouds, have demonstrated superior performance by enhancing the detection process, leading to more accurate and efficient 3D object detection models [7,18,19,20]. These advancements have pushed the boundaries of what is achievable in 3D object detection, particularly in dynamic and complex environments.

However, despite these advances, traditional 3D object detection methods are still constrained by the limitations of conventional object detection paradigms, particularly their reliance on annotated training datasets. This dependency restricts their ability to generalize to novel, previously unseen objects, making these models less effective in real-world applications where objects beyond the predefined classes may be encountered. In such complex environments, traditional detectors may struggle to recognize new objects, leading to poor performance.

### 2.2. Open-Vocabulary 2D Object Detection

Open-vocabulary 2D object detection is a task that aims to recognize and localize novel object classes that are not annotated in existing datasets [21]. This capability is essential for real-world applications, where the appearance of previously unseen objects can be frequent. The rapid development of large-scale image-text pre-training models has fueled significant progress in the field of open-vocabulary detection [22], enabling more flexible and accurate object recognition. One notable approach, RegionCLIP [23], integrates vision-language models to capture regional features and enhance category generalization, allowing the model to recognize objects outside the training set. Another method, Detic [24], selects the largest area proposals that cover the entire image, which helps to improve the model’s flexibility by utilizing large-scale pre-trained models for a broader range of objects. Additionally, GLIP [25] and MDETR [26] employ cross-modal pre-training techniques, leveraging both image and text modalities, which enhances their adaptability to complex scenes and diverse object categories.

Despite these advances in 2D open-vocabulary detection, this approach primarily focuses on 2D image space [8,17]. In contrast, this work focuses on the more challenging problem of open-vocabulary 3D object detection, which remains an underexplored area in current research. This is a critical task, especially for applications in autonomous driving and robotics, where recognizing and localizing unseen 3D objects is essential.

### 2.3. Open-Vocabulary 3D Object Detection

Open-vocabulary 3D object detection aims to recognize and localize novel object classes within 3D scenes using 3D bounding boxes, a task of great significance in complex environments. This field has attracted considerable attention in domains such as autonomous driving, robotics, and augmented reality due to its potential to address the challenge of recognizing previously unseen objects. Unlike traditional object detection methods that rely on annotated datasets, open-vocabulary 3D object detection seeks to identify objects that are not part of the training set, thereby enabling more flexible and robust performance in dynamic, real-world scenarios [27].

One approach to solving this problem involves leveraging pre-trained models from datasets like ImageNet-1K [6], which significantly enhances the localization and recognition capabilities of 3D models in open-vocabulary settings. By integrating the knowledge of multiple foundational models, these approaches improve the generalization ability of 3D detectors when dealing with novel object classes. Additionally, some methods make use of unlabeled data to generate pseudo-labels, thus expanding the training dataset and improving model performance. In these approaches, the model makes initial predictions, and high-confidence predictions are added to the training set as pseudo-labels, which helps enhance both localization accuracy and classification performance, especially for unseen objects [6,28].

More recent work in this area has seen the introduction of deep learning-based methods specifically tailored for open-vocabulary 3D object detection (OV-3Det) [29]. These methods employ the CLIP model, combined with a large-scale pre-trained external OV-2Det model, to generate synthetic labels for potential novel objects. Other approaches, such as CoDA, attempt to address the same problem but without relying on external OV-2Det models as priors for novel bounding box localization [15,30]. Despite these advancements, many of these models rely exclusively on point cloud data for inference, limiting their ability to achieve satisfactory classification accuracy for novel objects.

In contrast, our work proposes a novel 3D open-vocabulary detection framework that incorporates multimodal data, such as point clouds and images, to enhance both detection accuracy and the ability to classify previously unseen objects. By leveraging the complementary information from multiple data modalities, our approach improves the robustness of the model in dynamic environments, offering a more comprehensive solution to the challenges of open-vocabulary 3D object detection.

## 3. Methods

In this section, we present details of the structure of the proposed method. We first provide a comprehensive overview of the proposed architecture for open-vocabulary 3D object detection. It consists of two innovative mechanisms for locating and classifying objects. Afterward, we explain one of two mechanisms called Robust Feature Classifier, which effectively identifies new objects by learning the various robust features. Finally, the other mechanism called Cross-modal Collaboration is presented, which obtains the precise position of the object through copositioning of 2D and 3D cross-modality.

### 3.1. Framework Overview

**Figure 2:**
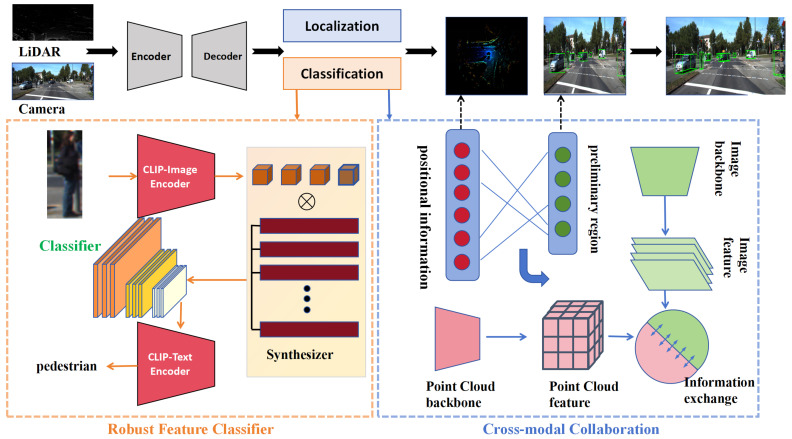
Overall frame of our method using Cross-modal Collaboration and Robust Feature Classifier. The former is responsible for localization, and the latter is responsible for classification in the process of object detection.

The overall architecture of our algorithm, Cross-modal Collaboration and Robust Feature Classifier, is illustrated in Figure 2. The core framework of our approach is 3DETR (3D Detection Transformer), which integrates the Transformer architecture with 3D object detection tasks. It adopts an end-to-end training paradigm, enabling direct object detection from point cloud data. Compared to other network architectures, our method does not rely on manually designed feature extractors, simplifying the pipeline while offering stronger generalization capabilities and the better handling of long-range dependencies. It demonstrates superior performance, particularly in sparse point clouds and occluded scenes. Functionally, our algorithm is divided into a localization module and a classification module. These correspond to our Cross-modal Collaboration module and Robust Feature Classifier module. The former fully leverages the advantages of multiple sensors to achieve more accurate positioning, while the latter enhances classification accuracy by enabling the classifier to learn robust features.

In the Robust Feature Classifier module, we utilize robust features generated by the synthesizer to train our classifier. To achieve the ability to recognize novel objects, we leverage the knowledge of CLIP as illustrated in Figure 2. This model has demonstrated significant potential in a wide range of tasks, such as image retrieval and search. After processing the training data through the CLIP encoding and synthesizer, they become a learnable robust feature, which are then fed into the classifier for training to achieve strong classification capabilities. During inference, the text embeddings output by the classifier are matched with the embeddings encoded by CLIP, ultimately yielding open-vocabulary categories as results.

In the Cross-modal Collaboration module, the 3D domain and 2D domain work together to localize novel objects. Initially, a 2D detector generates 2D bounding boxes from 2D detection images. Then, the 3D detection images are used to train a class-agnostic 3D detector, which generates class-agnostic 3D bounding boxes based on predicted camera parameters from the 2D detection images. Hungarian matching is subsequently performed between the 2D and 3D boxes to produce class-specific 3D bounding boxes. During this process, the 2D domain benefits from the precise location information provided by LiDAR, while the 3D domain gains clearer semantic information about the objects, such as color and shape, from the camera. After acquiring more accurate detection results, both domains provide refined information to each other, ultimately enabling the Cross-modal Collaboration to obtain the location information of novel objects.

### 3.2. Robust Feature Classifier

The Robust Feature Classifier module is designed to complement the training samples for untrained classes using the Contrastive Language–Image Pre-Training Model (CLIP). By synthesizing examples of untrained classes, this approach transforms the zero-shot classification problem into a conventional fully supervised problem, thereby alleviating the overfitting issue. However, it remains critical to design a method for generating features such that the trained classifier can accurately recognize and categorize novel objects. The main challenge lies in addressing the intra-class diversity and the inter-class similarity, which can lead to missed detection and confusion between classes. The Robust Feature Classifier is specifically designed to tackle these issues. It incorporates an intra-class diverging component to capture diversity within the same class and an inter-class preserving component to maintain clear distinctions between classes, enabling accurate classification.

**Figure 3:**
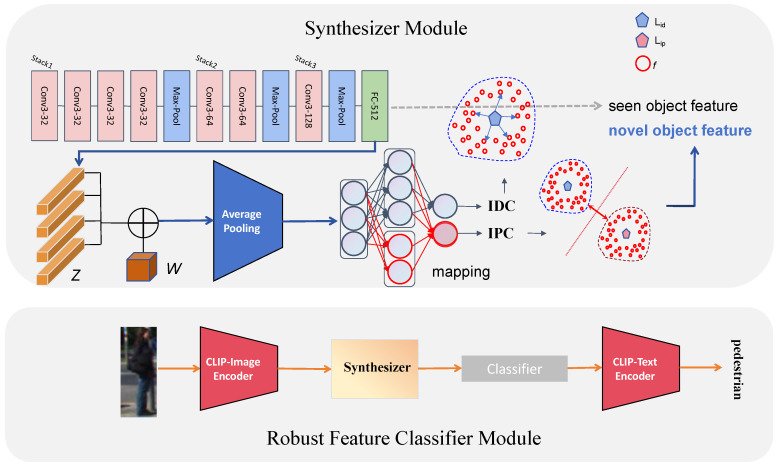
Workflow of Robust Feature Classifier. Its focus is on generating robust features that can be learned by the classifier, enabling it to achieve strong classification capabilities. Specifically, IDC and IPC refer to the intra-class diverging component and inter-class preserving component.

Figure 3 illustrates the proposed overall framework for the Robust Feature Classifier. The structure consists of two main parts: the recognition of known objects and the recognition of novel objects. The recognition of known objects follows the conventional object detection approach, where object features are automatically extracted. The network processes the raw image through multiple layers, learning low-level features such as edges, textures, and shapes, progressively constructing higher-level object representations for accurate identification and classification. For novel objects, which lack sufficient features, a feature synthesizer is required. This synthesizer includes components for the intra-class diverging component and inter-class preserving component.

Our Robust Feature Classifier extracts target features through a network consisting of convolutional layers, max-pooling layers, and fully connected layers. The convolutional layers are responsible for extracting high-level semantic features from the input feature maps. The convolution operation applies sliding windows to extract local information, capturing low-level features such as edges, textures, and shapes, and gradually builds more complex and abstract representations through multiple stacked convolutional layers. The max-pooling layers reduce the spatial resolution of the feature maps output by the convolutional layers, which helps enhance the translation invariance of features, ensuring that the network can still recognize objects even if their position changes within the image. The fully connected layers perform high-dimensional linear combinations of the pooled features, fully utilizing the global information of the input features for more accurate classification. The designed network structure effectively captures both local and global object features, making it especially suitable for multi-scale object recognition in target detection tasks. It progressively builds more precise object representations from details to the global level. Additionally, its streamlined structure reduces the size of the feature maps, alleviating computational burdens, while retaining important spatial information from the image. This is crucial for detecting objects with distinct spatial structures, such as pedestrians or vehicles.

The feature synthesizer, central to the Robust Feature Classifier, operates by learning a condition synthesizer S:W×Z→F, which maps class embeddings *w* and white noise vectors *z* to visual features *f* for detected regions for the class of interest. Using these synthesized features for untrained classes, a classifier can be trained for novel objects. Essentially, the generator learns the mapping from semantic vectors to their area-specific features. The function we propose is as follows:

(1) $$F_{o}=\lambda_{1}L_{c}+\lambda_{2}L_{id}+\lambda_{3}L_{ip}+\min_{S}\max_{D}L_{w}$$

The training objective for the synthesizer is defined by a function with hyper-parameters $\lambda_{1}$, $\lambda_{2}$ and $\lambda_{3}$ that balance different loss components. The term $L_{c}$ ensures that the visual features generated align with those of a pre-trained network on labeled data. To enhance robustness, two novel components, $L_{id}$ and $L_{ip}$, are introduced. Specifically, the former is the intra-class diverging loss, which spreads a distributed word representation into diverse region-level features, while the latter is the inter-class preserving loss, maintaining separability between synthesized features of different classes. The Wasserstein loss, $L_{w}$, encourages the synthesizer to synthesize region features that closely align with real ones:

(2) $$L_{w}=E\left[D\left(f,w\right)\right]-E\left[D\left(f^{\prime },w\right)\right]-\lambda E[{\left({\left||\nabla_{\hat{f}}D\left(\hat{f},w\right)|\right|}_{2}-1\right)}^{2}]$$

The above formula calculates the distance between feature vectors and maps the real features to the synthetic features, thereby enabling the computation of the loss function. And to generate multi-faceted visual representations, we introduce the intra-class diverging component. This approach mitigates the issue by increasing the influence of noise signals on the generated features while maintaining consistency with class-semantic embeddings. The class coherence loss ensures that features generated from adjacent noise signals are pulled together, while those from distinct vectors are pushed apart. This is achieved by selecting “positive” and “negative” pairs based on noise signals’ hemispheric location. Positive samples correspond to vectors from the positive hemisphere, while negative samples come from the negative hemisphere. The loss function is defined by cosine similarity between visual feature vectors, with τ as a temperature factor. Accordingly, the loss function is formulated as follows:

(3) $$L_{id}=E\left[-\log\frac{\exp(f^{\prime }\cdot f_{+}^{\prime }/\tau )}{\exp\left(f^{\prime }\cdot f_{+}^{\prime }/\tau \right)+\exp\left(f^{\prime }\cdot f_{-}^{\prime }/\tau \right)}\right]$$

To ensure the generated features resemble actual data while boosting discrimination, the inter-class preserving component is incorporated. This component uses both synthesized features from distinct classes and real features from proposed regions, including positive object proposals and negative background ones. It improves upon the traditional WGAN reconstruction error by aligning synthesized features with real ones of the same category within the proposal pool and pushing features from distinct classes further apart. The inter-class preserving loss ensures that synthesized visual features are close to both real and synthesized features of the same category while distant from features of other classes in the hybrid feature pool. The loss function of this component is as follows:

(4) $$L_{ip}=E\left[-\log\frac{\exp(f^{\prime }\cdot s_{+}/\tau )}{\exp\left(f^{\prime }\cdot s_{+}/\tau \right)+\sum_{i\in \Phi }\exp\left(f^{\prime }\cdot s_{i}/\tau \right)}\right]$$

In summary, the Robust Feature Classifier first extracts features from each candidate region in the input feature map, and then feeds the extracted class features into a classifier for learning. For novel objects, which lack sufficient features, we utilize the synthesizer to simulate real objects and generate relevant class features. To ensure that the generated features closely resemble the real features, we must ensure both the diversity and uniqueness of these features, which is the role of IDC and IPC.

### 3.3. Cross-Modal Collaboration

**Figure 4:**
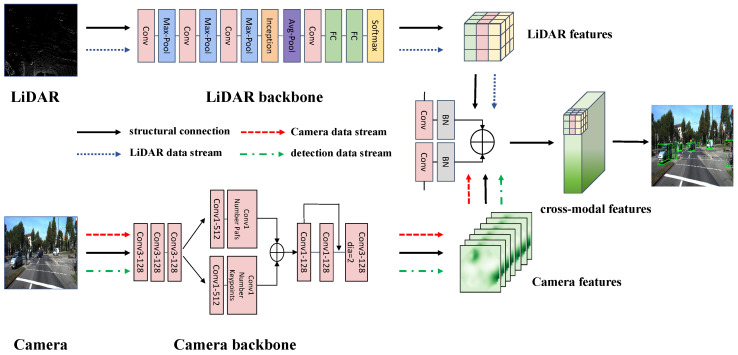
The proposed structure of Cross-modal Collaboration. We extract features for LiDAR and Camera. After converted into the same voxel space, they are added for the cross-modal features. The final result is obtained after cross-modal collaborative localization between these two modalities.

The Cross-modal Collaboration module leverages a multimodal architecture to integrate data from point clouds and images. This architecture supports cross-modal processing at test time, accommodating missing sensor inputs during inference. As Figure 4 shows, the structure consists of convolutional layers, max-pooling layers, average pooling layers, Inception modules, Softmax layers, and fully connected layers. The Inception module is used for multi-scale feature extraction, enabling the model to simultaneously capture spatial features at different resolutions. In a multimodal setting, the scale and structure of objects in flat images and spatial point clouds may differ. And the Inception module enhances the model’s ability to extract diverse features by applying convolutional filters of different sizes in parallel, thus capturing both fine-grained and coarse features. The dual-parallel network structure can simultaneously process both 3D point cloud data and 2D image data, and it also incorporates a data linking bridge structure responsible for cross-modal communication. This architecture allows the model to fully and simultaneously utilize information from both modalities, leading to more comprehensive and accurate object detection across various scenarios.

For the below formula, 3D point cloud features $F_{c}$ are obtained via a voxelized backbone, while two-dimensional image features $F_{i}$ are obtained through an image backbone. By fusing information from multiple sources, the model is better able to generalize to unseen data. By training both 2D and 3D data, the model is able to learn more general features, which can improve performance in new objects and diverse inspection scenarios. Image features are projected into the 3D grid space, generating $F_{i}^{\prime }$ using camera parameters. Specifically, 3D voxel positions are projected to the 2D image plane through the intrinsic matrix and extrinsic matrix, linking the two modalities for collaborative object detection. However, 3D point cloud data, rich in spatial information, are critical for detection but often overshadow image features during training. To mitigate performance degradation when point cloud data are absent at inference, we regularize features with 3D convolution and layer normalization, preventing feature suppression. A cross-modal training approach is then employed, where the detection network occasionally obtains features from $F_{m},F_{c},F_{i}^{\prime }$ with set probabilities. This ensures that 3D point cloud data and 2D image data are treated on an equal footing, facilitating the model’s ability to fully utilize the information from both modalities during Cross-modal Collaboration:

(5) $$F_{m}=LayerNorm\left(conv\left(F_{i}^{\prime }\right)\right)+LayerNorm\left(conv\left(F_{c}\right)\right)$$

Collaboration between modalities enhances detection. When 2D modalities assist 3D detection, 2D bounding boxes—leveraging abundant data and annotations—are projected into 3D space. Points within 2D boxes are clustered to generate corresponding 3D boxes, which can include novel objects absent in annotations. This enables profound semantic awareness from pre-trained 2D models to propagate into 3D, improving open-vocabulary detection. And when 3D modalities assist 2D detection, 3D boxes are generated independently at first and then communicate object position information with 2D image modality. During inference, these 3D boxes supplement predictions from the multimodal transformer, with overlapping boxes filtered using non-maximum suppression (NMS). The spatial information of the 3D inspection further pinpoints the pre-selected areas of the 2D inspection. Specifically, the pre-trained 3D detector’s confidence scores are scaled and assigned to 2D boxes, enhancing accuracy.

The Cross-modal Collaboration enables mutual assistance for detecting both two modalities objects, specifically, 2D boxes with four values and 3D boxes with seven values. In order to measure the detection effect of each iteration of the algorithm, we need to develop a proprietary loss function for the cross-modal coordination mechanism. The loss functions include L1 regularization with the Generalized IoU objective for 2D box localization, and L1 regularization with the separated IoU criterion in 3D regression tasks. This ensures that 3D point cloud data provide more accurate object boundaries and positions in situations where objects are partially occluded or the environment is cluttered, where 2D images can be difficult. The combination of the two can improve the detection accuracy of the model in complex or occluded environments. To address novel classes in open-vocabulary settings, uncertainty estimation μ is applied to L1 loss for regression, forming the detection loss function:

(6) $$L=\sqrt{2}\cdot \exp\left(-\mu \right)\cdot \left(L_{1}^{3D}+L_{cls}+L_{1}^{2D}\right)+L_{IoU}^{3D}+L_{IoU}^{2D}+\mu$$

In the formula, $L_{1}^{2D}$ and $L_{1}^{3D}$ represent the L1 loss functions in the 2D and 3D modes. The L1 loss function measures the absolute difference between predicted and ground truth values, promoting stability and robustness in regression tasks. It penalizes errors linearly, making it less sensitive to outliers compared to L2 loss, and is widely used in bounding box regression for object detection. $L_{cls}$ quantifies the difference between predicted class probabilities and ground truth labels. Commonly implemented as cross-entropy loss, it evaluates the effectiveness of a model in assigning correct labels to input samples and is fundamental in tasks like image classification or object detection. $L_{IoU}^{3D}$ and $L_{IoU}^{2D}$ are Intersection over Union losses in two modes. The IoU loss evaluates the overlap between predicted and ground truth bounding boxes, focusing on spatial alignment. Variants like GIoU or DIoU extend this to consider bounding box distance and size mismatches. IoU-based losses are essential in object detection to optimize box localization beyond just minimizing distances. Together, these loss functions enable accurate and well-localized object detection systems.

## 4. Experiments

### 4.1. Dataset

**Figure 5:**
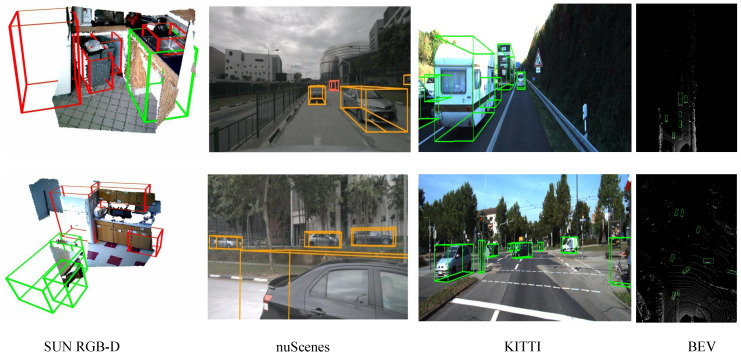
Visualization results of our method for open-vocabulary 3D detection on the SUN RGB-D, nuScenes, and KITTI datasets. These datasets encompass a variety of indoor and outdoor scenes. The last column shows the detection results in BEV (Bird’s Eye View) on the KITTI dataset. Green and orange represent base objects, while other colors represent novel objects.

As Figure 5 shows, we conduct extensive experiments under various conditions for both open-vocabulary and traditional closed-vocabulary settings to demonstrate the strong detection capabilities of our method, which leverages Cross-modal Collaboration and Robust Feature Classifier.

Specifically, the datasets are divided into indoor and outdoor categories [31,32]. The SUN RGB-D dataset is a widely used multimodal dataset for indoor scene understanding, designed to provide rich annotated data for tasks such as object detection, semantic segmentation, and 3D reconstruction in indoor environments. It includes RGB images, depth maps, and 3D point clouds captured by RGB-D cameras. Each image is annotated with object category labels and spatial location information. The dataset consists of approximately 10,000 images covering over 80 indoor object categories, including furniture, appliances, and office equipment. In addition to RGB and depth images, it also provides 3D point cloud data, which accurately capture the 3D structure and spatial relationships of objects [33].

KITTI, on the other hand, is an outdoor dataset designed for autonomous driving and robotic perception. It includes sensor data from various outdoor environments, such as stereo images, LiDAR point clouds, GPS, and IMU. Initially developed for autonomous vehicle perception systems, KITTI offers rich traffic scene data, including roads, traffic signs, pedestrians, and vehicles. The dataset provides over 15,000 frames of image data across urban streets, highways, and rural roads, with annotated object categories and spatial locations. The LiDAR point clouds in KITTI offer precise 3D object detection and localization, particularly in complex outdoor settings [15]. The nuScenes dataset, created by Aptiv and MIT, is a large-scale dataset focused on autonomous driving in urban and complex environments. Like KITTI, it includes multimodal data such as RGB images, LiDAR point clouds, radar, IMU, and GPS. nuScenes features approximately 1000 complete driving trajectories, each lasting about 20 s, with annotations for hundreds of object categories, including vehicles, pedestrians, and traffic signs [34]. It provides detailed 3D bounding boxes, object poses, and category labels, combining various sensor data to enhance object detection in challenging environments. In the experiment, we test the open vocabulary object detection capability of our model by using one part of these datasets as the base category and the other part as the unknown category.

### 4.2. Implementation Details

Our algorithm is primarily implemented using MMDetection3D and trained with the AdamW optimizer. We utilize the ‘mmdet3d.models’ module to implement several classical models for comparison experiments, and the ‘mmdet3d.datasets’ module to interface with the data pipeline. Additionally, we employ ‘mmdet3d.ops’ for basic operations such as point cloud processing, feature extraction, and convolution operations. The point cloud branch follows the same structure as Uni3DETR [35]. For the encoder–decoder architecture, we use tools from ‘torchvision.transforms’ for data augmentation operations, such as image rotation, cropping, and normalization, which help enhance the model’s generalization capability. We use ResNet50 and FPN as image feature extractors. The pre-trained ResNet50 model is loaded via ‘torchvision.models.resnet50’, and we fine-tune it on our task. The FPN module is implemented using ‘torchvision.models.detection.FPN’, which optimizes multi-scale feature extraction. The pre-trained Detic model [36] is used for 2D open-vocabulary detection, and all generated labels are filtered with a confidence threshold of 0.4. During training, we use the ‘torch.optim’ optimizer module to update model parameters. The proposed method is trained on two NVIDIA GeForce RTX 4090 GPUs (NVIDIA, Santa Clara, CA, USA) with a batch size of 8. Initially, a base 3DETR model is trained for 600 epochs using only class-agnostic distillation [37]. Subsequently, the base model is further trained for an additional 200 epochs incorporating the proposed Cross-modal Collaboration and Robust Feature Classifier. All experiments are conducted in the following environment: the operating system is Ubuntu 20.04, with CUDA version 11.3, Python version 3.9.8, PyTorch version 1.11.0, and MMDetection3D version 1.4.0.

### 4.3. Main Results

The performance of our method, which leverages Cross-modal Collaboration and Robust Feature Classifier, on the SUN RGB-D dataset for open-vocabulary 3D object detection surpasses all other methods. Our approach achieves superior results in both novel AP and basic AP metrics on this dataset. We first evaluate our method on the indoor SUN RGB-D dataset using the 46-class setting and report the AP metric in Table 1. All AP values in the table are computed with a confidence threshold of 0.25 [4]. Here, $AP_{novel}$ represents the detection accuracy for novel objects (i.e., classes not labeled in the training set), while $AP_{base}$ indicates the detection accuracy for base objects (i.e., classes labeled in the training set). CoDA, selected as our baseline, is also the state of the art (SOTA) in the field of open-vocabulary object detection. Our method achieves 14.49% $AP_{25}$ for the 36 novel classes using point clouds only during inference, surpassing CoDA by 7.44% under the same data conditions. This result demonstrates the strong ability of our method to recognize novel classes due to the Robust Feature Classifier. Additionally, our $AP_{base}$ is even 10.03% higher, further validating the classifier’s robust performance. We also observe that 3D-CLIP, another multimodal input method, performs worse than CoDA in our experiments. In contrast, our multimodal approach outperforms CoDA, demonstrating that our Cross-modal Collaboration effectively integrates multimodal knowledge and comprehensively utilizes information from different modalities. This integration significantly contributes to achieving superior performance in open-vocabulary 3D object detection.

We then evaluate our method on the outdoor KITTI dataset. For simplicity during training, we do not use true labels sampling augmentation [40]. We report the $AP_{25}$ (the confidence threshold is 0.25) metric with 11 recall positions for moderate difficulty objects from the validation set, with the results presented in Table 2. Outdoor point clouds, typically collected by LiDAR sensors, often contain a high proportion of background points, with foreground objects being small, sparse, and containing significantly fewer points. Using single-modal point cloud inputs inevitably results in a significant performance drop. For multimodal inputs, although the additional camera modality provides useful information, the noisy data from 3D point clouds may still contaminate the detection results. These challenges make 3D detection of novel objects in outdoor scenes particularly difficult. Despite this, our method achieves 24.08% $AP_{25}$ for the novel class in outdoor scenes, surpassing CoDA by 8.30%. For the multimodal results, $AP_{novel}$ is further improved to 15.17%. Similarly, for the nuScenes dataset, our method outperforms the advanced CoDA and the multimodal 3D-CLIP across all metrics. This validates the superiority of our Cross-modal Collaboration and Robust Feature Classifier in both localization and classification tasks. Moreover, it demonstrates the ability of our method to detect objects in both indoor and outdoor scenes, thus achieving scene unification.

**Figure 6:**
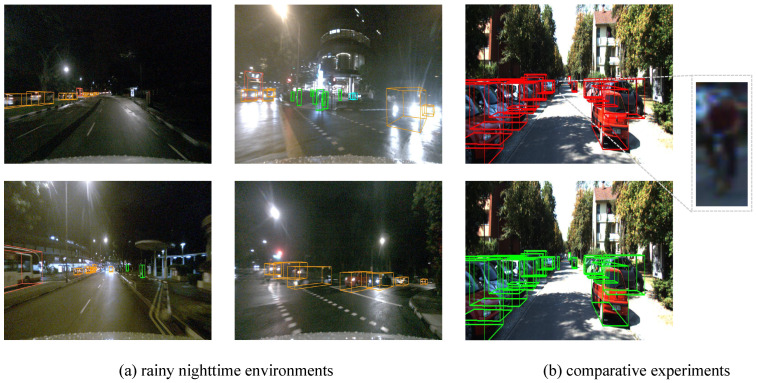
Visualization results of our method for rainy nighttime environments and comparative experiments. (**a**) The detection performance of our model under rainy night conditions. Orange represents base objects, and other colors represent novel objects. (**b**) Comparison experiments, where red represents SOTA method, and green represents ours.

Then, we evaluate our method in the traditional closed-vocabulary 3D detection setting. Although the superiority of our method has been validated under open-vocabulary conditions, it is also necessary to assess its performance in closed-vocabulary datasets to ensure the algorithm’s general applicability in practical scenarios. We evaluate on the KITTI dataset and compare our results with previous 3D detection methods on the test set [10]. The AP values with a confidence threshold of 0.4 are presented in Table 3. As shown, our method achieves state-of-the-art performance across all categories (car, pedestrian, and cyclist) and difficulty levels (easy, moderate, and hard). The three difficulty levels of the KITTI dataset, “Easy,” “Mod.” (Moderate), and “Hard,” are primarily defined based on object visibility, size, and occlusion degree. Easy means that objects are clearly visible, relatively large in size, with a simple background and minimal occlusion. Mod. means that objects are partially occluded, of moderate size, and situated in more complex backgrounds. Hard means that objects are heavily occluded, small in size, with complex backgrounds, making detection more challenging. These difficulty levels reflect the complexity of different detection scenarios and are used to evaluate the performance of algorithms under various conditions. Compared to CLOCs (the current SOTA), our method achieves 3D AP improvements for the car category by 2.99%, 2.53%, and 1.19% at the easy, moderate, and hard levels, respectively. Additionally, in 2D performance, our method also surpasses CLOCs in BEV AP, with improvements of 1.63%, 1.98%, and 1.53% for the respective difficulty levels. Considering both the robustness of the model in various complex environments and its competitiveness compared to other state-of-the-art (SOTA) models, we conduct experiments on datasets from challenging environments, such as rainy nights, and perform comparisons with SOTA methods. As shown in Figure 6, our model still achieves promising detection results despite adverse conditions such as rain and nighttime. In the comparison with SOTA, our method successfully detects a cyclist that is missed by the SOTA approach in the same scene. This clearly demonstrates the superior detection capability of our model for small, distant, and non-vehicle objects. These results validate that our method exhibits strong generalization and detection capabilities, regardless of the application scenario or the objects being detected.

### 4.4. Ablation Study

This subsection presents ablation studies that analyze the contributions of individual modules—Cross-modal Collaboration and Robust Feature Classifier—to the performance of 3D object detection. We also report the combined effect of these modules. The experiments highlight the superiority of the proposed enhancements over the baseline model. Note that our ablation study is conducted on the KITTI Validation Set, with 3D-CLIP as the baseline model. Since our method is primarily designed to address the needs of the autonomous driving domain, we choose the KITTI dataset, which is rich in road data, for the ablation experiments. To control for the experimental variables, we select the 3D-CLIP model with multimodal inputs as the baseline to ensure consistency in the training dataset conditions.

As shown in Table 4, both the Cross-modal Collaboration and Robust Feature Classifier modules significantly improve the detection accuracy. When added individually to the baseline, there is a notable increase in $AP_{25}$ for both unseen classes (pedestrian) and visible classes (car, cyclist). Specifically, when only Cross-modal Collaboration is included, the AP improvements for the three target categories are 7.54%, 12.64%, and 11.19%, respectively. When only the Robust Feature Classifier is added, the improvements are 8.72%, 18.03%, and 7.81%. Furthermore, when both modules are combined, we observe a further significant improvement in accuracy compared to using either module individually. This is because the two modules complement each other; although both aim to enhance object detection accuracy, Cross-modal Collaboration operates at the localization stage, while the Robust Feature Classifier focuses on the classification stage. Their combined effect results in a synergistic improvement rather than a dilution of benefits, leading to a substantial increase in AP, further validating the superiority of the two proposed algorithmic modules.

### 4.5. Computational Efficiency

To enhance the computational efficiency of 3D object detection, our model employs some lightweight techniques such as fast convolutions and multi-scale feature interaction decoupling. The former helps reduce redundant computations during feature extraction, while the latter accelerates the multimodal collaboration process. We then conduct comparative experiments with other open-vocabulary object detection methods. As shown in the Table 5, although our algorithm has lower computational efficiency compared to single-modality LiDAR-based methods, it demonstrates a significant improvement in computational speed when compared to the multimodal algorithm 3D-CLIP.

Considering the high real-time requirements in autonomous driving applications, single-frame inference typically needs to be completed within 100 ms. This is because the refresh rate of most LiDARs or cameras ranges from 10 to 30 Hz, meaning the maximum processing time per frame is approximately 33–100 ms. Our processing time of 89 ms meets the minimum requirement. However, to ensure rapid vehicle response to the environment, the inference time must be significantly lower than the maximum time interval between sensor refreshes. Therefore, our future research will focus on further improving computational efficiency. We aim to optimize detection speed by pursuing model lightweighting. We plan to explore methods such as knowledge distillation, pruning, and quantization to reduce the computational load and enhance inference speed. Furthermore, we will design efficient feature extraction algorithms and optimize multimodal data fusion strategies to reduce cross-modal computational overhead, enabling fast and accurate object detection. We will also consider designing execution strategies for hardware accelerators like GPU and TPU to improve parallel computation and inference efficiency.

## 5. Conclusions

In this paper, we introduce Cross-modal Collaboration and Robust Feature Classifier for open-vocabulary 3D object detection. The former helps the model accurately locate objects, while the latter aids in correct classification. The mechanism of Cross-modal Collaboration involves collaborative localization between the 2D camera and 3D LiDAR modalities. During this collaboration, 3D spatial information and 2D color–texture information are fused across modalities, leading to more precise object localization. Therefore, the key feature of Cross-modal Collaboration is to maximize the integration of multimodal information in the case of multimodal inputs, fully leveraging the advantages of multi-sensor perception. On the other hand, the Robust Feature Classifier is designed to specifically address two major challenges in novel object classification: misidentifying objects as background and misclassifying them as other objects. Its focus is on a feature synthesizer combining intra-class diverging component and inter-class preserving component. The classifier learns robust features from these components to gain powerful classification ability, overcoming the difficulties in classification and ensuring correct object categorization. In summary, the combination of the Cross-modal Collaboration mechanism and the Robust Feature Classifier mechanism excels at accomplishing open-vocabulary 3D object detection tasks.

We then conducted extensive experiments to validate the superiority of our algorithm. To ensure the generalizability of the results, we tested our method on multiple scenarios and multi-source datasets, including both indoor and outdoor scenes. The datasets used include SUN RGB-D, nuScenes, and KITTI. We compared our method with several advanced approaches, including both single-modality and multimodality input models. The results show that our method outperforms others in terms of detection accuracy across all object categories. Moreover, whether for open-vocabulary or closed-vocabulary detection, our method achieves the best performance. Finally, we conducted ablation experiments to verify the effectiveness of individual modules, with results indicating that the two proposed modules significantly improve object detection accuracy. Additionally, when combined, they exhibit even better performance, further validating the theoretical advantages of our modules in localization and classification.

In summary, our method, which employs Cross-modal Collaboration and Robust Feature Classifier, demonstrates state-of-the-art performance both theoretically and practically. It has significant implications for advancing autonomous driving technology and driving innovation in the field of 3D perception.

## Figures and Tables

**Table 1:** The performance of our method, which leverages Cross-modal Collaboration and Robust Feature Classifier, on the SUN RGB-D dataset for open-vocabulary 3D object detection surpasses all other methods. Our approach achieves superior results in both $AP_{novel}$ and $AP_{base}$ metrics on this dataset. The bold numbers are the best results in the comparison test.

Modality	Method	SUN RGB-D		
		$AP_{novel}$(%)	$AP_{base}$(%)	$AP_{all}$(%)
LiDAR	PointCLIP [27]	0.13	5.17	1.32
	PointCLIPv2 [32]	0.21	4.72	1.28
	Det-CLIP2 [38]	1.07	23.57	6.02
	OV-3DET [29]	5.85	38.24	12.77
	CoDA [6]	7.05	41.23	14.96
LiDAR + RGB	3D-CLIP [39]	4.20	32.33	10.16
	Ours	**14.49**	**51.26**	**22.11**

**Table 2:** The performance of our method, which leverages Cross-modal Collaboration and Robust Feature Classifier, on the KITTI and nuScenes datasets dataset for open-vocabulary 3D object detection surpasses all other methods. Our approach achieves superior results in both $AP_{novel}$ and $AP_{base}$ metrics on this dataset. The bold numbers are the best results in the comparison test.

Modality	Method	KITTI			nuScenes		
		$AP_{Ped}$(%)	$AP_{Car}$(%)	$AP_{Cyc}$(%)	$AP_{novel}$(%)	$AP_{base}$(%)	$AP_{all}$(%)
LiDAR	PointCLIP	0.20	8.56	3.11	0.37	8.79	5.19
	PointCLIPv2	0.43	9.50	5.75	0.59	9.06	5.25
	Det-CLIP2	2.16	40.75	20.18	3.16	51.61	29.89
	OV-3DET	10.84	77.29	37.03	9.36	49.16	25.51
	CoDA	15.78	84.00	40.54	13.78	55.13	30.54
LiDAR + RGB	3D-CLIP	8.91	66.54	35.10	10.39	52.90	27.22
	Ours	**24.08**	**91.76**	**53.16**	**22.17**	**66.34**	**39.76**

**Table 3:** The performance of our method used Cross-modal Collaboration and Robust Feature Classifier on KITTI test for closed-vocabulary 3D object detection. Our method outperforms all the other methods in both 3D AP and BEV AP metrics. The bold numbers are the best results in the comparison test.

Class	Modality	Method	3D AP(%)			BEV AP(%)		
			Easy	Mod.	Hard	Easy	Mod.	Hard
Car	LiDAR	FPRCNN [13]	74.79	68.26	60.17	80.09	76.16	70.37
		VRCNN [15]	78.43	71.97	63.17	84.16	80.14	75.62
		PVRCNN [19]	79.44	73.19	65.02	86.75	81.56	78.24
	LiDAR + RGB	3dCVF [41]	80.88	71.56	63.24	88.14	80.46	72.15
		FConv [42]	84.16	76.06	66.14	89.15	81.66	75.23
		Transfusion [18]	86.25	78.91	70.83	90.15	82.23	75.80
		CLOCs [43]	88.07	77.81	70.11	90.45	82.01	76.24
		Ours	**91.06**	**80.34**	**71.30**	**92.08**	**83.99**	**77.77**
Ped	LiDAR	FPRCNN	42.76	35.49	30.79	50.14	45.26	38.19
		VRCNN	46.45	40.71	35.19	51.60	46.97	42.07
		PVRCNN	50.12	40.16	34.78	61.45	52.79	46.89
	LiDAR + RGB	3dCVF	53.44	46.06	39.99	65.47	60.72	53.71
		FConv	56.67	49.81	43.15	68.79	60.18	56.62
		Transfusion	59.04	52.11	42.18	75.04	70.16	62.18
		CLOCs	61.82	51.97	45.10	78.41	71.16	63.40
		Ours	**62.04**	**54.39**	**46.32**	**80.01**	**72.93**	**64.05**
Cyc	LiDAR	FPRCNN	60.17	49.73	43.17	62.19	50.19	47.26
		VRCNN	65.17	51.47	46.28	65.79	53.16	49.69
		PVRCNN	68.03	51.79	49.23	70.25	61.33	55.74
	LiDAR + RGB	3dCVF	68.21	59.14	50.41	70.14	59.94	54.62
		FConv	73.52	61.11	55.49	75.05	64.50	60.75
		Transfusion	76.10	62.26	57.27	76.98	65.22	60.64
		CLOCs	78.82	64.31	59.33	78.90	69.22	63.64
		Ours	**80.09**	**65.37**	**60.95**	**80.17**	**70.06**	**64.25**

**Table 4:** The ablation study conducted on the KITTI dataset. CC is Cross-modal Collaboration and RFC is Robust Feature Classifier. As before, the car and cyclist classes are seen during training, while the pedestrian class is novel. The bold numbers are the best results in the comparison test.

Modality	CC	RFC	KITTI		
			$AP_{Ped}$(%)	$AP_{Car}$(%)	$AP_{Cyc}$(%)
LiDAR + RGB	×	×	8.19	66.54	35.10
	✓	×	15.73	79.18	46.29
	×	✓	16.91	84.57	42.91
	✓	✓	**24.08**	**91.76**	**53.16**

**Table 5:** The computational efficiency of our algorithm compared to other open-vocabulary object detection algorithms on the KITTI dataset. ‘Times’ in the table refers to the inference time per frame. ‘L’ means LiDAR and ‘L + R’ means LiDAR + RGB.

	PointCLIP	PointCLIPv2	Det-CLIP2	OV-3DET	CoDA	3D-CLIP	Ours
Modality	L	L	L	L	L	L + R	L + R
Times (ms)	66	73	70	75	75	97	89

## Data Availability

nuScenes dataset at https://www.nuscenes.org/. KITTI dataset at https://www.cvlibs.net/datasets/kitti/ (accessed on 19 December 2024).
